# Supplementary material for: Effects of Linseed Meal and Carotenoids from Different Sources on Egg Characteristics, Yolk Fatty Acid and Carotenoid Profile and Lipid Peroxidation
Source: Foods. 2021 May 31;10(6):1246. doi: 10.3390/foods10061246 (PMC8226844; doi:10.3390/foods10061246)
Supplement: Supplementary file 1 [file foods-10-01246-s001.zip › foods-1201573-supplementary.pdf]

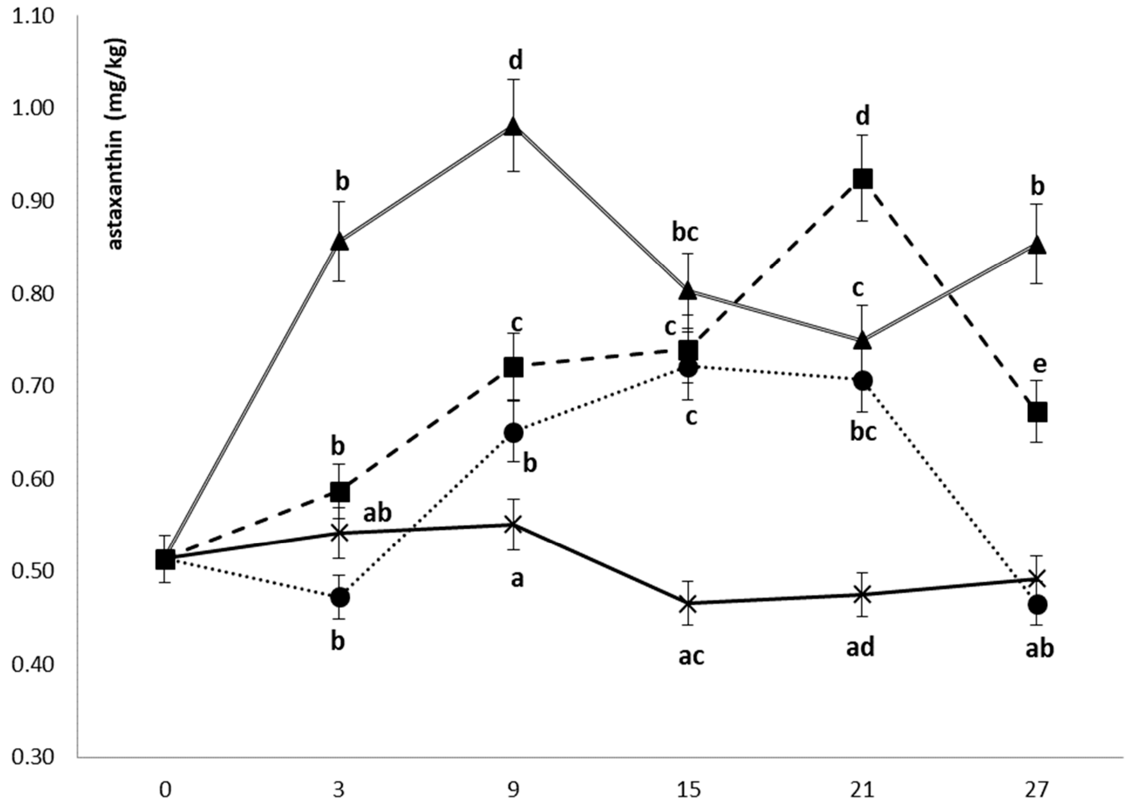

Figure S1. Time evolution (0–27 days) of yolk astaxanthin concentration according to the dietary treatments: control (●), experimental E1(■), experimental E2 (▲) and experimental E3 (×). C—control diet; E1—diet supplemented with 6% linseed meal + 2% dried kapia pepper; E2—diet supplemented with 6% linseed meal + 2% dried sea buckthorn pomace; E3—diet supplemented with 6% linseed meal + 2% dried carrot.

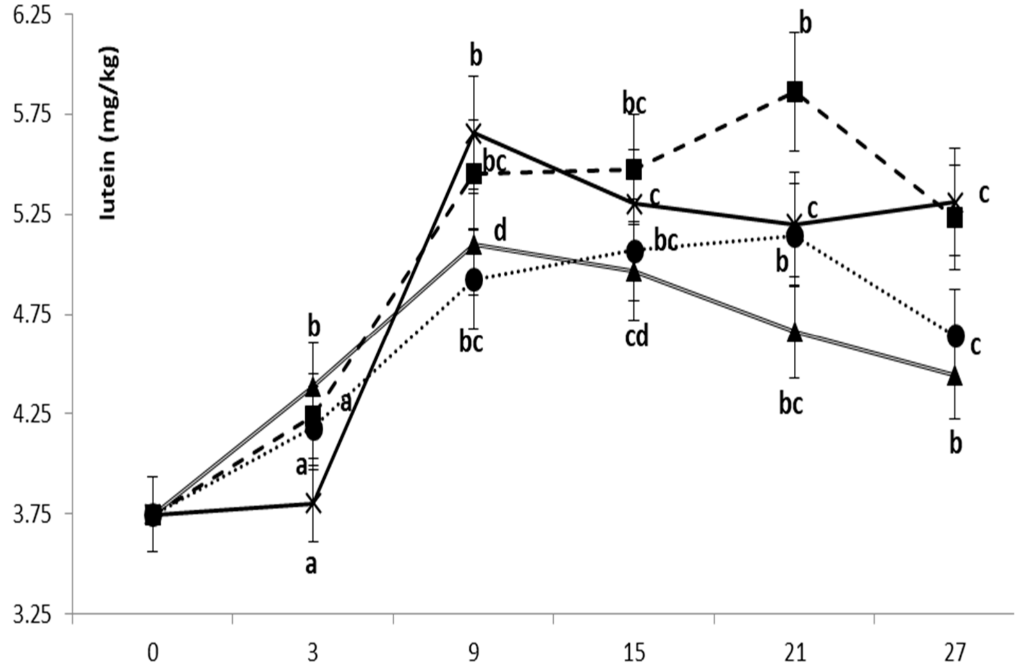

Figure S2. Time evolution (0–27 days) of yolk lutein concentration according to the dietary treatments: control (●), experimental E1(■), experimental E2 (▲) and experimental E3 (×). C—control diet; E1—diet supplemented with 6% linseed meal + 2% dried kapia pepper; E2—diet supplemented with 6% linseed meal + 2% dried sea buckthorn pomace; E3—diet supplemented with 6% linseed meal + 2% dried carrot.

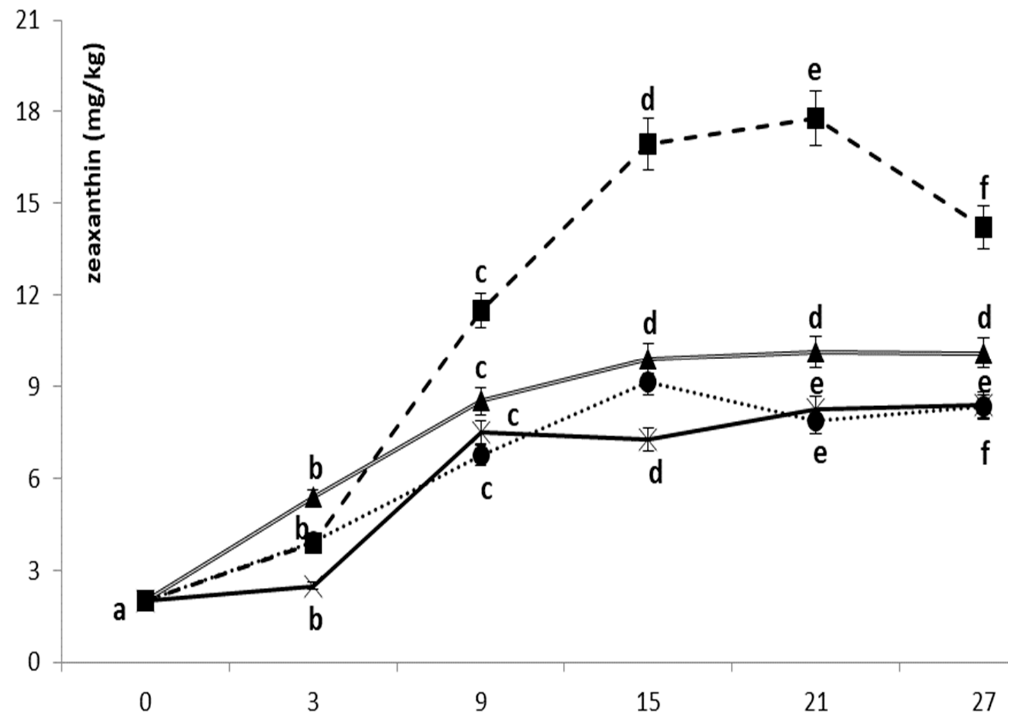

Figure S3. Time evolution (0–27 days) of yolk zeaxanthin concentration according to the dietary treatments: control (●), experimental E1(■), experimental E2 (▲) and experimental E3 (×). C—control diet; E1—diet supplemented with 6% linseed meal + 2% dried kapia pepper; E2—diet supplemented with 6% linseed meal + 2% dried sea buckthorn pomace; E3—diet supplemented with 6% linseed meal + 2% dried carrot.

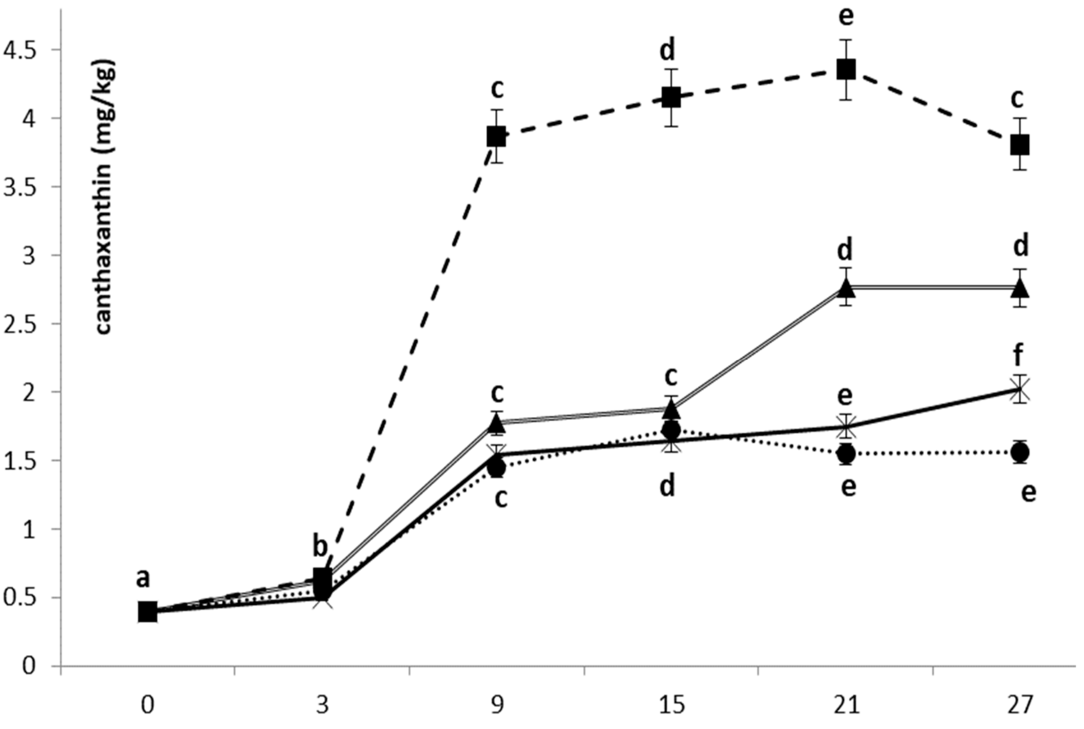

Figure S4. Time evolution (0–27 days) of yolk canthaxanthin concentration according to the dietary treatments: control (●), experimental E1(■), experimental E2 (▲) and experimental E3 (×). C—control diet; E1—diet supplemented with 6% linseed meal + 2% dried kapia pepper; E2—diet supplemented with 6% linseed meal + 2% dried sea buckthorn pomace; E3—diet supplemented with 6% linseed meal + 2% dried carrot.

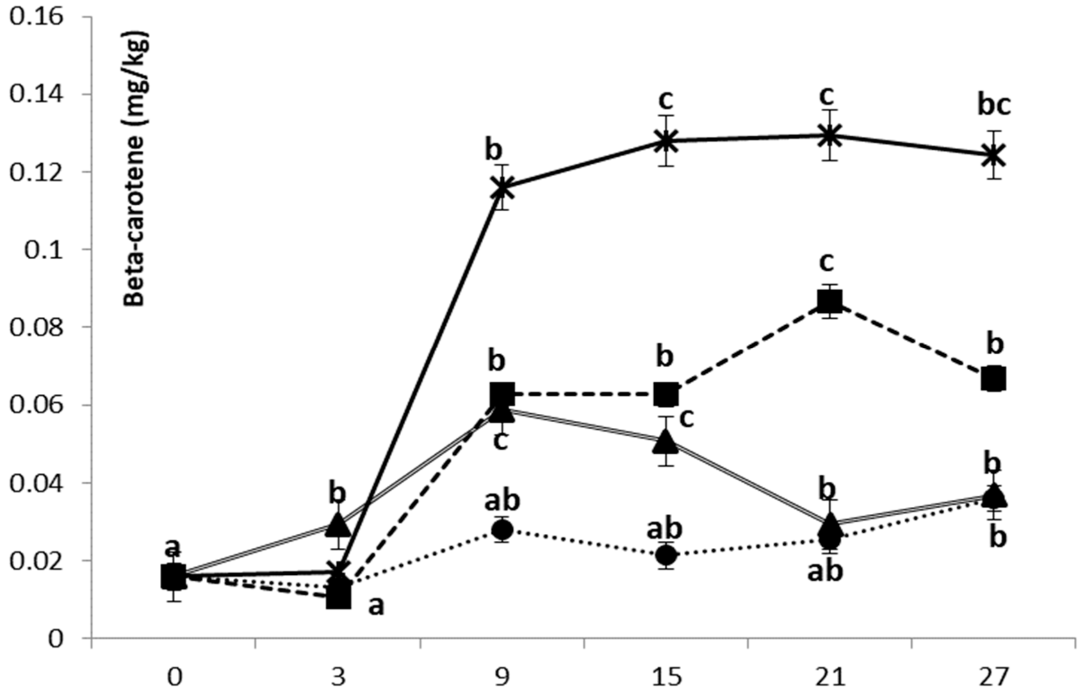

Figure S5. Time evolution (0–27 days) of yolk beta-carotene concentration according to the dietary treatments: control (●), experimental E1(■), experimental E2 (▲) and experimental E3 (×). C—control diet; E1—diet supplemented with 6% linseed meal + 2% dried kapia pepper; E2—diet supplemented with 6% linseed meal + 2% dried sea buckthorn pomace; E3—diet supplemented with 6% linseed meal + 2% dried carrot.

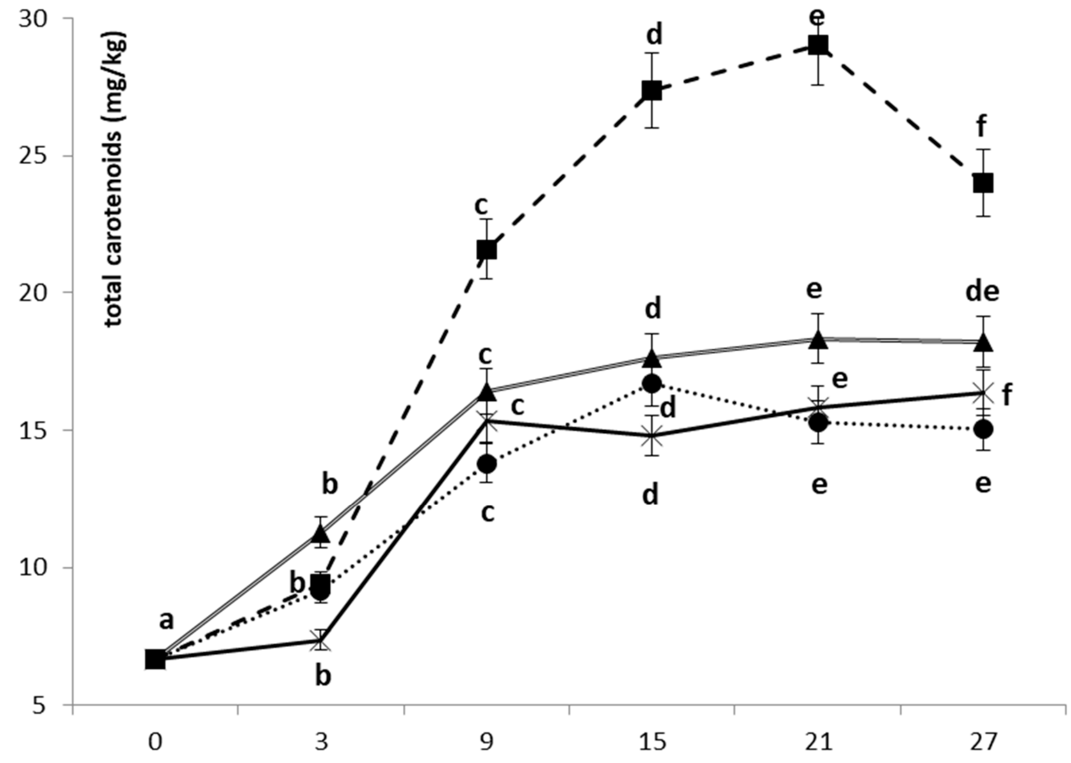

Figure S6. Time evolution (0–27 days) of yolk total carotenoids concentration according to the dietary treatments: control (●), experimental E1(■), experimental E2 (▲) and experimental E3 (×). C—control diet; E1—diet supplemented with 6% linseed meal + 2% dried kapia pepper; E2—diet supplemented with 6% linseed meal + 2% dried sea buckthorn pomace; E3—diet supplemented with 6% linseed meal + 2% dried carrot.
